# The Role of Nephrologists in the Management of Methanol Poisoning

**DOI:** 10.7759/cureus.37471

**Published:** 2023-04-12

**Authors:** Yassine Allata, Basmat Amal Chouhani, Ghita El bardai, Nadia Kabbali, Tarik Sqalli Houssaini

**Affiliations:** 1 Nephrology, Hemodialysis and Transplantation, Hassan II University Hospital, Fez, MAR; 2 Epidemiology and Health Sciences Research Laboratory, Faculty of Medicine, Pharmacy and Dentistry, Sidi Mohamed Ben Abdellah University, Fez, MAR

**Keywords:** anion gap, adulterated alcohol, dialysis, metabolic acidosis, methanol intoxication

## Abstract

Acute methanol poisoning is a rare but serious condition that can lead to significant morbidity and mortality. Toxic metabolites produced by methanol, primarily formaldehyde, can cause high anion gap metabolic acidosis, with the severity of clinical presentation ranging from mild symptoms to multi-organ failure. Nine people died and four patients needed treatment at our university hospital following a collective intoxication caused by the consumption of homemade alcoholic beverages in the central region of Morocco. The four patients presented to the emergency department with varying clinical symptoms, such as decreased visual acuity, severe agitation, and dyspnea. The laboratory tests confirmed high anion gap metabolic acidosis and a subsequent toxicology screen revealed that they had consumed methanol-tainted alcohol. The treatment regimen involved inhibiting the formation of toxic metabolites using an antidote (ethanol or fomepizole), correcting metabolic acidosis, enhancing the elimination of toxic metabolites through prolonged hemodialysis, and administering adjunctive therapies. While two patients had favorable outcomes, the other two died from multi-organ failure. These findings highlight the importance of prompt diagnosis and treatment in cases of methanol poisoning.

## Introduction

Methanol, also known as wood alcohol or methyl alcohol, is a clear, colorless, and flammable liquid with a sweet, sugary smell. It is produced by the distillation of wood or through chemical synthesis from natural gas or coal. Methanol is used in many industrial processes, including the production of formaldehyde, acetic acid, and methyl tert-butyl ether (MTBE), as well as in the manufacture of biodiesel and as a solvent in the paint and plastics industries. It is also used as a fuel for race cars, boats, and airplanes, as it burns cleaner than gasoline. Methanol is a popular alternative fuel source because it is renewable, abundant, and can be produced from a variety of sources [1].

Methanol is a highly toxic substance, and exposure to it can cause significant harm to human health. Methanol can be absorbed through inhalation, ingestion, or skin contact. Inhalation of methanol vapor can cause eye, nose, and throat irritation, as well as headache, dizziness, and nausea. Ingestion of even small amounts of methanol can cause serious harm, including blindness, coma, and death. The toxicity occurs through the metabolism of methanol to formaldehyde and then to formic acid. Formaldehyde is a highly reactive and toxic substance that can cause damage to DNA and proteins. Formic acid is an even more toxic substance that can cause metabolic acidosis, blindness, and neurological damage. The mechanism of ocular toxicity is not fully understood, but formic acid is known to play an important role in central nervous system depression [2].

The symptoms of methanol poisoning can vary depending on the amount and route of exposure. The poisoning can occur accidentally, such as through inhalation in the workplace or skin exposure with the use of alcohol rubs in children. It can also result from consuming alcohol that is contaminated with methanol, using burning alcohol that contains high levels of methanol, or consuming homemade spirits.

The clinical presentation of methanol poisoning is characterized by a latency phase before the onset of specific toxic effects related to its metabolites. The latency phase can range from a few hours to several days, depending on the amount and route of exposure. The symptoms typically include headache, dizziness, nausea, and vomiting. In severe cases, it can cause metabolic acidosis, which is characterized by an increase in the anion gap and a decrease in bicarbonate levels. Metabolic acidosis can lead to hyperventilation, which can cause Kussmaul-type breathing (deep, rapid breathing).

The ocular effects of methanol poisoning can include blurred vision, decreased visual acuity, and blindness. The mechanism of ocular toxicity is not fully understood, but it is believed to be caused by formic acid, which can accumulate in the retina and optic nerve [3].

In Morocco, methanol poisoning outbreaks are not uncommon and often result in multiple deaths. In this article, we report on a collective methanol poisoning incident that occurred in a small village, resulting in nine deaths and the hospitalization of four others. We present four cases of methanol poisoning who presented to our hospital after the incident and describe their clinical presentation, management, and outcomes.

## Case presentation

Patient A

A 21-year-old man with a history of chronic alcoholism presented to the emergency department complaining of decreased visual acuity and blurred vision a few hours after consuming adulterated alcohol. Upon admission, he was disoriented with a Glasgow Coma Scale score of 13. The patient's hemodynamics were stable with a blood pressure of 110/60 mmHg, but he exhibited Kussmaul-type expiratory dyspnea. An hour after admission, the patient became agitated and rapidly deteriorated, necessitating intubation and mechanical ventilation. An arterial blood gas analysis revealed severe metabolic acidosis, with a pH of 7.11, a PaCO_2_ of 11 mmHg, and a bicarbonate level of 3.8 mmol/L, consistent with high anion gap metabolic acidosis. The osmolar gap was not measured due to unavailability at our hospital. Urgent prolonged hemodialysis was initiated along with the administration of 1.4% sodium bicarbonate serum at a dose of 250 mL intravenously every four hours, and blood gas levels were monitored twice daily. On the second day, toxicological results were still not available, but there was a strong suspicion of methanol poisoning. As such, we administered the appropriate antidote, medical ethanol, due to the unavailability of fomepizole in our country, with a loading dose of 0.6 g/kg followed by a maintenance dose of 100 mg/kg/hour. Despite the cessation of sedation, patient A did not show any signs of improvement by the fourth day of treatment, and an MRI revealed ischemic lesions with limbic necrosis and early tonsillar herniation. Unfortunately, the patient suffered an irreversible cardiac arrest on the fifth day.

Patient B

A 33-year-old man presented to the emergency department with acute dyspnea. On admission, the patient was conscious, with a Glasgow Coma Scale score of 15, a blood pressure of 130/92 mmHg, and expiratory dyspnea. Laboratory analysis revealed high anion gap metabolic acidosis with a pH of 7.3, a PaCO_2_ of 14 mmHg, and bicarbonate of 5 mmol/L. The electrolyte panel showed no other significant abnormalities. The patient received a dialysis session on the same day and was subsequently placed on the same treatment protocol as patient A. The patient was subsequently transferred to the nephrology department and was discharged on the fourth day with a normal physical examination and blood workups.

Patient C

A 35-year-old man with no significant medical history was admitted to the emergency department by the ambulance service for severe agitation. On admission, the patient was unstable, with a blood pressure of 90/50 mmHg and signs of peripheral hypoperfusion. Despite resuscitative measures, the patient suffered a cardiac arrest in the resuscitation room and could not be revived.

Patient D

A 30-year-old man with a history of chronic alcoholism and regular cannabis use was admitted to the emergency department with vomiting and minor hematemesis 24 hours after consuming adulterated alcohol. On examination, the patient was conscious, with a blood pressure of 130/78 mmHg, a heart rate of 110 beats/min, pale, and had Kussmaul-type expiratory dyspnea. Laboratory analysis revealed high anion gap metabolic acidosis with bicarbonate of 5.6 mmol/L, a pH of 7.24, and a PaCO_2_ of 18 mmHg. The rest of the laboratory findings were unremarkable (Table 1).

**Table 1:** Laboratory test results for admitted patients. BUN: blood urea nitrogen; PaCO_2_: partial pressure of carbon dioxide; PaO_2_: partial pressure of oxygen.

Laboratory tests	Patient A	Patient B	Patient D
Creatinine (mg/dL)	0.9	0.8	1.1
BUN (mg/dL)	190	100	280
Serum sodium (mmol/L)	137	133	136
Serum potassium (mmol/L)	3	4.6	3.4
Serum chloride (mmol/L)	102	108	102
Bicarbonate (mmol/L)	4	5	6
pH	7.11	7.3	7.24
PaCO_2_ (mmHg)	11	14	18
PaO_2_ (mmHg)	142	139	135
Anion gap (mmol/L)	33	20	28

The patient received a dialysis session on admission and was subsequently started on the same treatment protocol as the other two patients, along with a proton pump inhibitor. Twice daily arterial blood gas analysis showed a gradual improvement in the acidosis, and after two days, the patient's laboratory values returned to normal, prompting the discontinuation of dialysis and ethanol treatment protocol. The patient was kept under observation for an additional two days and was finally discharged with a normal physical examination, blood workups, and renal function.

## Discussion

**Figure 1:**
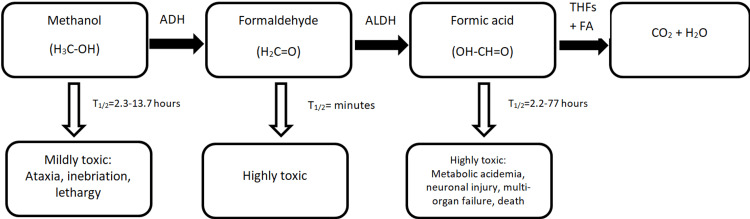
Metabolism and clinical manifestations of methanol poisoning. ADH: alcohol dehydrogenase; ALDH: aldehyde dehydrogenase; THFs: 5,10-methylenetetrahydrofolate; FA: folic acid; T_1/2_: half-life.

When ingested, methanol is metabolized by the liver to form toxic metabolites, including formaldehyde and formic acid. These metabolites are responsible for the toxic effects of methanol (Figure 1).

The delay in seeking medical attention can contribute significantly to the high mortality rate associated with methanol poisoning. This was particularly evident in this incident where 11 people died because of methanol poisoning. Out of the 11 fatalities, nine individuals died at home, while the other two died in the hospital. The reluctance to seek medical help stems from various reasons, including shame associated with consuming illegal alcohol, limited access to healthcare facilities, and being inebriated.

The management of methanol poisoning requires a multidisciplinary approach, involving emergency physicians, toxicologists, and nephrologists. The first step in managing methanol poisoning is to ensure that the patient's airway, breathing, and circulation are stable. This may involve providing oxygen, administering intravenous fluids, and monitoring the patient's vital signs. In severe cases, mechanical ventilation and vasopressors may be required to support the patient's respiratory and cardiovascular systems [4].

Once the patient's immediate needs have been addressed, treatment for methanol poisoning typically involves administering an antidote called fomepizole or ethanol. Both of these agents work by inhibiting the metabolism of methanol, allowing the body to eliminate it more slowly and reducing the production of toxic metabolites. Fomepizole is the preferred first-line treatment for methanol poisoning due to its superior efficacy in preventing the formation of toxic metabolites. Administered intravenously, it is given as a loading dose followed by maintenance doses every 12 hours until the body eliminates methanol [5]. However, its high cost limits its availability, particularly in low and middle-income countries. In contrast, ethanol, which is a cheaper alternative, has been used for many years as an antidote for methanol poisoning. It is available orally at a concentration of 20% and injectable at a concentration of 10% and is typically administered as a continuous infusion until methanol has been eliminated. Ethanol works by competing with methanol for the same metabolic pathways, effectively slowing down the production of toxic metabolites.

In addition to administering an antidote, other supportive measures may be necessary to manage the complications of methanol poisoning such as renal replacement therapy (RRT) to remove methanol and its toxic metabolites from the blood. Intermittent hemodialysis is considered the most effective RRT modality for managing methanol poisoning, reducing the duration of antidotal treatment, and shortening the hospital observation period [6]. The formation of metabolites before antidote administration limits the efficacy of the antidotes, and hemodialysis is required to remove the toxin and its metabolites and correct metabolic acidosis (Table 2) [7,8].

**Table 2:** Toxicokinetic summary of the effect of extracorporeal treatments on the elimination of methanol.

Type of extracorporeal treatment	Methanol Clearance (mL/min)	Methanol T_1/2 _(hours)
Intermitant hemodialysis	208 (77–400)	3.4 (0.6–13.1)
Peritoneal dialysis	37 (5–70)	13 (2–49)
Continuous renal replacement therapy	36.7 (17–48)	8.6 (3.5–12)

Adjuvant therapies include the administration of folic acid, which acts as a cofactor in the final breakdown of formic acid in methanol poisoning. It is administered intravenously at a dose of 50 mg every four hours for 24 hours [4].

The prognosis for methanol poisoning depends on a variety of factors, including the dose of methanol ingested, the time elapsed since ingestion, and the promptness and effectiveness of treatment. If left untreated, methanol poisoning can lead to serious complications such as blindness, central nervous system depression, and even death [9]. If there is suspicion of methanol or other harmful alcohol poisonings, initiating RRT rapidly, although not a toxin-specific treatment, is the most effective way to remove the toxin and its toxic metabolites, prevent end-organ damage, and minimize adverse central nervous system sequelae, as methanol can cause permanent neurological damage [10].

Prevention of methanol poisoning is key and involves both education and regulation. Education efforts should focus on raising awareness of the dangers of methanol, particularly in regions where it is commonly used as an alternative to ethanol or as an adulterant in illicit alcohol. Regulation of methanol-containing products, such as windshield washer fluid and antifreeze, can also help reduce the incidence of methanol poisoning.

## Conclusions

This case report highlights the urgent need for early detection and intervention in cases of methanol poisoning. Delay in seeking medical attention is a major problem that can lead to devastating consequences. Dialysis plus ethanol treatment was found to be effective in managing the condition and is relatively low cost compared to other treatment options. However, further studies with larger sample sizes and control groups are necessary to confirm its effectiveness. The report underscores the importance of educating the public and regulating the sale and distribution of illicit alcohol to prevent methanol poisoning.
